# Assessment of thiol-disulfide and glutathione homeostasis after levothyroxine replacement in individuals with autoimmune or nonautoimmune hypothyroidism

**DOI:** 10.20945/2359-4292-2023-0197

**Published:** 2024-08-05

**Authors:** Almila Senat, Osman Erinc, Soner Yesilyurt, Gamze Gok, Ozcan Erel

**Affiliations:** 1 Taksim Training and Research Hospital Istanbul Türkiye Taksim Training and Research Hospital, Medical Biochemistry, Istanbul, Türkiye; 2 Taksim Training and Research Hospital Istanbul Türkiye Taksim Training and Research Hospital, Internal Medicine, Istanbul, Türkiye; 3 Ankara City Hospital Ankara Türkiye Ankara City Hospital, Medical Biochemistry, Ankara, Türkiye

**Keywords:** Glutathione, Hashimoto’s thyroiditis, hypothyroidism, thiol-disulfide homeostasis, oxidative stress

## Abstract

**Objective:**

Thyroid hormones are known to affect the biosynthesis and degradation of antioxidant compounds, suggesting a possible link between hypothyroidism and oxidative stress. However, there is no clear consensus in the literature regarding this association. The aim of this study was to evaluate oxidative stress markers (extracellular thiol-disulfide homeostasis and intracellular glutathione homeostasis) in patients with hypothyroidism due to autoimmune (Hashimoto’s thyroiditis) or nonautoimmune thyroid disease rendered euthyroid after levothyroxine replacement.

**Subjects and methods:**

The study included 116 patients admitted to the Taksim Training and Research Hospital (Istanbul, Türkiye). Of these, 50 had hypothyroidism due to Hashimoto’s thyroiditis (HT group), 30 had nonautoimmune hypothyroidism (NAIH group), and 36 were healthy controls (control group). All participants were women. Extracellular thiol-disulfide homeostasis and intracellular glutathione homeostasis tests were assessed as oxidative stress markers.

**Results:**

Thiol-disulfide homeostasis in both HT and NAIH groups was shifted toward the oxidative spectrum. Compared with the control group, the HT and NAIH groups had lower levels of native (p < 0.001 and p = 0.001, respectively) and total (p = 0.002 and p = 0.012, respectively) thiol, as well as a lower native thiol/total thiol ratio (p < 0.001 for both). The HT group also had higher disulfide levels than the control group (p = 0.027). Reduced glutathione (GSH) and oxidized glutathione (GSSG) values were comparable across all three groups, but the HT and NAIH groups had higher GSSG/GSH (p < 0.001 for both) and GSSG/(GSH+GSSG) ratios (p = 0.003 and p = 0.005, respectively), along with lower GSH/(GSH+GSSG) ratio (p = 0.001 and p = 0.002, respectively) than the control group.

**Conclusion:**

Levothyroxine replacement was ineffective in ameliorating oxidative stress in patients with hypothyroidism due to Hashimoto’s thyroiditis or nonautoimmune causes, as extracellular thiol-disulfide homeostasis was notably altered in these patients compared with healthy controls. The findings of this study suggest that oxidative stress remains a prevailing issue in patients with autoimmune or nonautoimmune hypothyroidism even after euthyroidism is restored.

## INTRODUCTION

Thyroid hormones play a crucial role in various metabolic processes, and imbalances in their levels are among the most prevalent endocrine disorders (1,2). Hypothyroidism, a hypometabolic condition resulting from decreased synthesis and secretion of thyroid hormones, affects approximately 12% of the population. It can be categorized into nonautoimmune and autoimmune types, based on the presence or absence of thyroid antibodies. Nonautoimmune hypothyroidism is most commonly seen after administration of certain medications (*e.g.*, amiodarone, thalidomide, rifampin, ethionamide, phenobarbital) and thyroid surgery, or may occur as central hypothyroidism secondary to pituitary or hypothalamic inflammatory, genetic, or neoplastic disorders (3,4). In subclinical hypothyroidism, serum thyroid-stimulating hormone (TSH) levels are elevated, but free thyroxine (fT^4^) levels remain normal. In overt hypothyroidism, TSH levels are high, while fT4 levels are reduced (5,6). Autoimmune thyroid conditions arise from a dysfunction in the immune system, causing it to target the thyroid gland. The primary clinical presentations of autoimmune thyroid disorders are predominantly Hashimoto’s thyroiditis and Graves’ disease (7).

Hashimoto’s thyroiditis, a type of autoimmune hypothyroidism, is the most common thyroid disorder among women. This condition is marked by the infiltration of T and B lymphocytes into the thyroid gland and the generation of thyroid autoantibodies, which eventually disrupt thyroid function (8,9). In Hashimoto’s thyroiditis, inflammation can lead to the destruction of thyroid tissue and damage to thyroid follicles (10). Thyroid hormones have been shown to influence the biosynthesis and degradation of antioxidant compounds, implying a plausible association between hypothyroidism and oxidative stress. Hypothyroidism has the potential to elevate the concentration of reactive species while concurrently diminishing antioxidant capacity, potentially precipitating oxidative stress (11).

Thiol molecules are compounds containing a sulfhydryl (-SH) group, which makes them inherently susceptible to oxidation. Extracellular thiol-disulfide homeostasis serves as a metric for protein thiol oxidation and provides insight into the levels of thiol and disulfide species. Maintaining this equilibrium is pivotal for a myriad of biological processes, including regulating protein functionality, preserving protein structural integrity, safeguarding cysteine residues from irreversible oxidation, facilitating chaperone functions, and modulating enzymatic activities and transcription (12).

Glutathione, one of the most essential intracellular antioxidants, plays a critical role in cellular metabolism. The ratio of oxidized glutathione (GSSG) to reduced glutathione (GSH) is a physiological signaling ratio that reflects cellular redox equilibrium. Numerous studies in the literature have evaluated intracellular glutathione homeostasis in various diseases (13,14).

The most recent findings on the relationship between thyroid hormone abnormalities and oxidative stress have been conflicting. Therefore, the aim of this study was to evaluate oxidative stress using the markers extracellular thiol-disulfide homeostasis and intracellular glutathione homeostasis in individuals with autoimmune or nonautoimmune hypothyroidism who were rendered euthyroid with levothyroxine replacement, compared with healthy controls.

## SUBJECTS AND METHODS

All study procedures involving human participants were conducted in accordance with the ethical standards of the institution and the national research committee, following the 1964 Helsinki Declaration and its subsequent amendments or comparable ethical standards. The study protocol was approved by the Clinical Research Ethics Committee of the Gaziosmanpasa Training and Research Hospital (Approval No. 03.15.2023/37).

The study included 116 patients admitted to the Taksim Training and Research Hospital (Istanbul, Türkiye), categorized into groups according to laboratory test results: 30 patients with nonautoimmune hypothyroidism, characterized by the absence of thyroid antibodies (*i.e.*, antithyroglobulin [anti-TG] and antithyroperoxidase [anti-TPO] antibodies), who were rendered euthyroid with regular use of levothyroxine (NAIH group); 50 patients with hypothyroidism due to Hashimoto’s thyroiditis, characterized by the presence of thyroid autoantibodies, who were also euthyroid due to levothyroxine use (HT group); and 36 healthy, sex-matched participants (control group). All participants were women.

**Figure 1:**
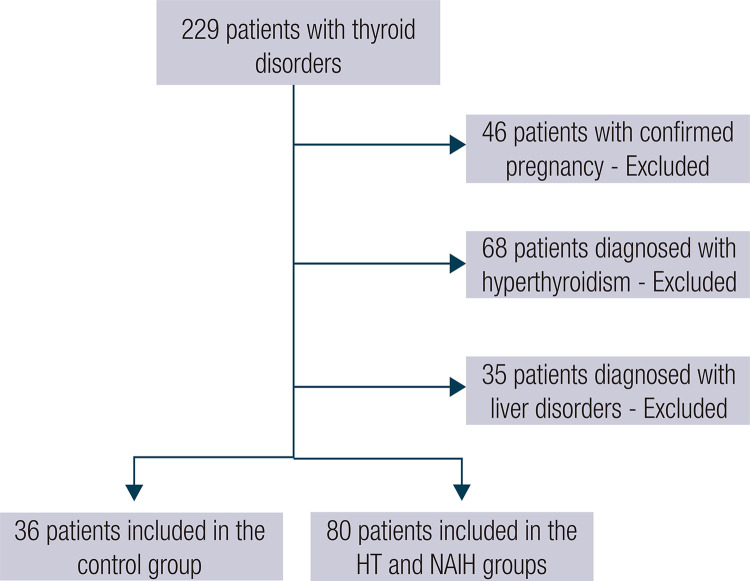
Flowchart of participant entry into the study.

A power analysis was conducted to determine the sample size for the present study, taking into account the results of previous studies. The calculated sample size was 107, using an impact size of 0.8, a significance level of 0.05, and a statistical power of 0.90. The exclusion criteria were a diagnosis of other metabolic or neurodegenerative diseases, pregnancy, and age below 18 years. Figure 1 shows a flowchart of participant entry into the study.

The diagnosis of Hashimoto’s thyroiditis was based on the presence of serum thyroid antibodies (anti-TG and/or anti-TPO) along with evidence of parenchymal heterogeneity on thyroid ultrasonography. Overt hypothyroidism was characterized by fT4 levels below the normal range (9.3-17 ng/dL) and TSH levels above the normal range (0.27-4.2 mIU/L). Upon receiving the diagnosis of hypothyroidism, the patients were started on levothyroxine replacement, with doses progressively adjusted according to TSH levels. The patients also received instructions on the proper use and storage of levothyroxine tablets.

Serum levels of TSH, free triiodothyronine (fT3), fT4, anti-TG, and anti-TPO were measured by chemiluminescence immunoassay on a Cobas e601 analyzer (Roche Diagnostics, Mannheim, Germany). Liver and kidney function tests were determined using spectrophotometric methods, and C-reactive protein was measured using an immunoturbidimetric method on a Cobas c501 analyzer (Roche Diagnostics).

Serum and whole blood samples from each participant were obtained in the morning following at least 8 hours of fasting. Blood samples in serum-separated tubes were centrifuged at 1,500 × g for 10 minutes. The supernatant fractions were aliquoted as serum samples and stored at -80°C. These samples were used for the assessment of extracellular thiol-disulfide homeostasis, which was done using a spectrophotometric method (15). Briefly, native thiol levels were measured using 5,5′-dithiobis(2-nitrobenzoic) acid (DTNB), a well-known thiol chromogen. Subsequently, the disulfide content of the sample was reduced to native thiol with the use of sodium borohydride. Formaldehyde was used to neutralize the excess sodium borohydride. Subsequently, the contents of both native thiols and reduced thiols were determined using the same thiol chromogen, providing a measurement of the total thiol levels. Disulfide levels were estimated by subtracting half of the native thiol concentrations from the total thiol concentration. The disulfide/native thiol, disulfide/total thiol, and native thiol/total thiol ratios (represented in percentages) were calculated from native thiol, total thiol, and disulfide levels.

Whole blood samples were collected into tubes containing ethylenediaminetetraacetic acid (EDTA) for assessment of intracellular glutathione homeostasis, following the preparation of erythrocyte lysate. These samples were centrifuged at 1,500 × g for 10 minutes, and the plasma fractions were decanted. After plasma separation, blood cells were washed three times using a 0.9% sodium chloride solution. When the washing steps were complete, the cells were lysed in distilled water. Three parts of cell lysate and one part of a 20% weight/volume trichloroacetic acid solution were used to precipitate proteins. The cell lysate precipitated with the trichloroacetic acid solution was centrifuged at 1,500 × g for 10 minutes and the supernatant fractions were stored at -80°C. These samples were used for the assessment of intracellular glutathione homeostasis, which was done using the semiautomated method described by Alisik and cols. (16). In this method, reduced glutathione (GSH) and total glutathione, which comprises GSH plus oxidized glutathione (GSH+GSSG), are measured in the supernatant fraction obtained from whole blood. The GSSG levels were calculated by subtracting the GSH content from the total glutathione value (GSH+GSSG) and dividing it by two. After obtaining the GSH, total GSH, and GSSG values, glutathione homeostasis was measured as GSSG/GSH, GSSG/total GSH, and GSH/total GSH ratios (represented in percentages).

### Statistical analysis

The statistical analyses were conducted using the software SPSS, Version 23.0 (IBM Corp., Armonk, NY, USA). The distribution of variables was assessed by the Kolmogorov-Smirnov test. Normally distributed variables were presented as mean ± standard deviation, while nonnormally distributed variables were represented as median (interquartile range). Categorical variables were expressed as percentages. The Kruskal-Wallis test and analysis of variance (ANOVA) were used to compare variables. The directional relationship between variables was assessed using Pearson’s and Spearman’s correlation tests. Multivariate regression analysis was performed to elucidate the potential impact of certain clinical and demographic characteristics on extracellular thiol-disulfide homeostasis and intracellular glutathione homeostasis in all participants. All comparisons were two-tailed, and the statistical significance was defined as a p value < 0.05.

## RESULTS

Table 1 summarizes the participants’ demographic and clinical characteristics, and Table 2 shows the results of the extracellular thiol-disulfide homeostasis analysis of serum samples. The results indicated significantly lower native thiol and total thiol levels in the NAIH and HT groups and increased disulfide levels in the HT group compared with the control group.

**Table 1:** Demographic and clinical characteristics of study participants

	Control group (n = 36)	NAIH group (n = 30)	HT group (n = 50)	P values
Age (years)	38.9 ± 14.4	52.25 ± 14.7	46.7 ± 13.7	**0.002**
Smoking (packs/year)	0 (0-0)	0 (0-15)	0 (0-20)	0.058
Duration of hypothyroidism (months)	–	71.6 ± 24.6	69.3 ± 21.7	0.911
Systolic blood pressure (mmHg)	120 (100-130)	120 (110-138)	120 (100-130)	0.547
Diastolic blood pressure (mmHg)	80 (65-80)	80 (70-90)	80 (65-80)	0.744
Diabetes mellitus (%)	0 (0)	3 (10)	3 (6)	0.097
Hypertension (%)	2 (5.5)	8 (26.6)	14 (28)	**0.010**
Coronary artery disease (%)	1 (2.7)	0 (0)	0 (0)	0.359
Cerebrovascular disease (%)	0 (0)	0 (0)	1 (2)	0.563
Peripheral artery disease (%)	0 (0)	0 (0)	1 (2)	0.563
Weight (kg)	61.5 (55-76.5)	82.6 (74.1-88.5)	70.4 (62.2-79.5)	**<0.001**
Height (cm)	159 ± 0.07	157 ± 0.05	157 ± 0.08	0.201
Body mass index (kg/m2)	25.6 ± 5.93	29.3 ± 12.1	26.5 ± 8.45	0.279
Creatinine (mg/dL)	0.63 ± 0.09	0.69 ± 0.11	0.68 ± 0.10	0.027
eGFR (mL/min/1.73 m2)	113 ± 14.2	97.4 ± 17.6	102 ± 17.7	**0.001**
Aspartate aminotransferase (U/L)	16 (14-19.7)	17.5 (16-22.2)	17 (15-22.5)	0.174
Alanine aminotransferase (U/L)	13 (9-19)	20.5 (14.5-25)	14.5 (10.7-21.2)	**0.013**
C-reactive protein (mg/dL)	2.16 ± 2.88	4.41 ± 5.54	3.61 ± 5.02	0.157
ESR (mm/hour)	7.02 ± 6.70	12.3 ± 7.39	14.4 ± 15.1	**0.018**
Free triiodothyronine (ng/dL)	3.17 ± 0.45	2.64 ± 0.29	2.86 ± 0.48	**<0.001**
Free thyroxine (ng/dL)	11.6 ± 1.21	11.03 ± 2.91	11.9 ± 2.44	0.237
TSH (mIU/L)	1.84 (1.29-2.83)	2.66 (1.90-3.88)	3.10 (2.13-4.03)	**0.001**
Anti-TG (kU/L)	14.3 (12.3-17.7)	14.3 (12.6-16.7)	196 (54.1-398)	**<0.001**
Anti-TPO (kU/L)	9.84 (9-13.7)	9 (9-9.36)	180 (55.7-307)	**<0.001**
Hemoglobin (g/L)	128 (117-133)	136 (129-141)	128 (114-134)	**0.007**

Values are shown as mean ± standard deviation or median (interquartile range). Abbreviations: anti-TG, antithyroglobulin antibodies; anti-TPO, antithyroperoxidase antibodies; eGFR, estimated glomerular filtration rate; ESR, erythrocyte sedimentation rate; TSH, thyroid-stimulating hormone.

**Table 2:** Extracellular thiol-disulfide homeostasis results in the study participants

	Control group (n = 36)	NAIH group (n = 30)	HT group (n = 50)	P values
Native thiol (µmol/L)	382 ± 48.8	325 ± 58.7	338 ± 58	* **<0.001** ** = **0.001** *** = 0.717
Total thiol (µmol/L)	423 ± 52.5	369 ± 63.4	385 ± 61.1	* = **0.002** ** = **0.012** *** = 0.497
Disulfide (µmol/L)	20.4 ± 3.44	23.6 ± 5.37	23.7 ± 6.95	* = 0.094 ** = **0.027** *** = 0.997
Disulfide/native thiol (%)	5.37 ± 0.85	7.51 ± 1.98	7.17 ± 2.28	* <**0.001** ** <**0.001** *** = 0.741
Disulfide/total thiol (%)	4.84 ± 0.69	6.48 ± 1.48	6.20 ± 1.71	* < **0.001** ** < **0.001** *** = 0.707
Native/total thiol (%)	90.3 ± 1.38	87.1 ± 2.97	87.5 ± 3.43	* < **0.001** ** < **0.001** *** = 0.709

Values are shown as mean ± standard deviation. *Control group versus NAIH group. **Control group versus HT group. ***NAIH group versus HT group. Abbreviations: NAIH, nonautoimmune hypothyroidism; HT, Hashimoto’s thyroiditis.

Table 3 displays the results of the intracellular glutathione homeostasis analysis in erythrocyte lysate. No significant differences in GSH, total GSH (GSH+GSSG), or GSSG values were observed across all three groups (p > 0.05 for all). However, the GSSG/GSH and GSSG/(GSH+GSSG) ratios were significantly higher and the GSH/(GSH+GSSG) ratio significantly lower in the NAIH and HT groups compared with the control group.

**Table 3:** Intracellular glutathione homeostasis results in the study participants

	Control group (n = 36)	NAIH group (n = 30)	HT group (n = 50)	P values
GSH (µmol/L)	613 (560-650)	613 (598-753)	613 (550-732)	* = 0.334 ** = 0.355 *** = 0.949
GSH+GSSG (µmol/L)	139 ± 44.1	156 ± 35.7	153 ± 45.2	* = 0.334 ** = 0.355 *** = 0.949
GSSG (µmol/L)	42.2 ± 18.1	53.2 ± 16.3	51.1 ± 19.2	* = 0.095 ** = 0.106 *** = 0.896
GSSG/GSH (%)	60.4 (53.9-78.2)	133 (85.5-140)	108 (83.6-130)	* < **0.001** ** < **0.001** *** = 0.149
GSSG/(GSH+GSSG) (%)	29.4 ± 4.41	33.3. ± 3.86	32.5 ± 3.87	* = **0.003** ** = **0.005** *** = 0.698
GSH/(GSH+GSSG) (%)	41.1 ± 8.82	33.2 ± 7.73	34.9 ± 7.75	* = **0.001** ** = **0.002** *** = 0.421

Values are shown as mean ± standard deviation or median (interquartile range). *Control group versus NAIH group. **Control group versus HT group. ***NAIH group versus HT group. Abbreviations: GSH, reduced glutathione; GSSG, oxidized glutathione; HT, Hashimoto’s thyroiditis group; NAIH, nonautoimmune hypothyroidism group.

The results of the directional relationships between thyroid function tests (including anti-TG, anti-TPO, TSH, fT3, and fT4) and extracellular thiol-disulfide homeostasis and intracellular glutathione homeostasis assessments are shown in Table 4.

**Table 4:** Directional relationship between oxidative stress indicators and thyroid function tests

	Anti-TG	Anti-TPO	TSH	fT3	fT4
Native thiol (µmol/L)	**r = -0.230** **p = 0.017**	r = -0.108 p = 0.267	**r = -0.280** **p = 0.004**	**r = 0.415** **p < 0.001**	r = -0.027 p = 0.786
Total thiol (µmol/L)	**r = -0.242** **p = 0.011**	r = -0.101 p = 0.297	**r = -0.253** **p = 0.009**	**r = 0.397** **p < 0.001**	r = 0.061 p = 0.533
Disulfide (µmol/L)	r = 0.150 p = 0.119	r = -0.043 p = 0.654	r = 0.122 p = 0.212	r = -0.046 p = 0.636	r = -0.023 p = 0.812
GSH (µmol/L)	r = -0.043 p = 0.676	r = -0.028 p = 0.785	r = 0.100 p = 0.337	r = 0.135 p = 0.195	r = -0.081 p = 0.439
GSH+GSSG (µmol/L)	r = 0.023 p = 0.823	r = 0.091 p = 0.380	r = 0.088 p = 0.398	r = -0.095 p = 0.360	r = -0.141 p = 0.174
GSSG (µmol/L)	r = 0.050 p = 0.629	r = 0.190 p = 0.291	r = 0.061 p = 0.556	r = -0.143 p = 0.169	r = -0.136 p = 0.191

Abbreviations: anti-TG, antithyroglobulin antibodies; anti-TPO, antithyroperoxidase antibodies; fT3, free triiodothyronine; fT4, free thyroxine; GSH, reduced glutathione; GSSG, oxidized glutathione; TSH, thyroid-stimulating hormone.

The results of the multivariate regression analysis examining the impact of specific clinical and demographic characteristics on homeostasis assessments are shown in Table 5. Age was the only variable with a significant influence on native (adjusted R^2^ 0.560, p < 0.001) and total (adjusted R^2^ 0.523, p < 0.001) thiol levels. Conversely, weight, body mass index, and hypertension had no significant effect on either extracellular thiol-disulfide or intracellular glutathione homeostasis assessments (all p > 0.05).

**Table 5:** Results of multivariate regression analysis examining the impact of clinical and demographic characteristics on thiol-disulfide homeostasis and glutathione homeostasis assessments

	uSt. B	95% confidence intervals	P values
**Native thiol**			
Age (years)	-2.643	(-3.30) – (-1.98)	**<0.001**
Hypertension (%)	-15.1	(-39.05) – (8.71)	0.211
Body mass index (kg/m^2^)	-0.767	(-1.99) – (0.454)	0.216
Weight (kg)	-0.048	(-0.191) – (0.095)	0.507
Adjusted R2 0.560, **p < 0.001**			
**Total thiol**			
Age (years)	-2.78	(-3.50) – (-2.06)	**<0.001**
Hypertension (%)	-7.96	(-33.6) – (17.7)	0.540
Body mass index (kg/m^2^)	-0.979	(-2.27) – (0.318)	0.137
Weight (kg)	-0.059	(-0.213) – (0.096)	0.452
Adjusted R2 0.523, **p < 0.001**			
**Disulfide**			
Age (years)	-0.045	(-0.143) – (0.053)	0.366
Hypertension (%)	1.88	(-1.62) – (5.38)	0.289
Body mass index (kg/m^2^)	0.016	(-0.161) – (0.193)	0.857
Weight (kg)	0.003	(-0.018) – (0.024)	0.809
Adjusted R^2^ 0.024, p = 0.823			
**GSSG/GSH ratio**			
Age (years)	-0.200	(-0.785) – (0.385)	0.499
Hypertension (%)	25.2	(3.79) – (46.6)	0.022
Body mass index (kg/m^2^)	-0.150	(-1.15) – (0.855)	0.767
Weight (kg)	0.068	(-0.05) – (0.188)	0.262
Adjusted R^2^ 0.058, p = 0.055			
**GSSG/(GSH+GSSG) ratio**			
Age (years)	-0.031	(-0.105) – (0.043)	0.412
Hypertension (%)	3.31	(0.590) – (6.04)	**0.018**
Body mass index (kg/m^2^)	-0.027	(-0.155) – (0.101)	0.676
Weight (kg)	0.005	(-0.011) – (0.020)	0.548
Adjusted R^2^ 0.042, p = 0.099			
**GSH/(GSH+GSSG) ratio**			
Age (years)	0.062	(-0.087) – (0.210)	0.412
Hypertension (%)	-6.62	(-12.1) – (-1.18)	**0.018**
Body mass index (kg/m^2^)	0.054	(-0.202) – (0.309)	0.418
Weight (kg)	-0.009	(-0.04) – (0.021)	0.677
Adjusted R^2^ 0.042, p = 0.099			

Abbreviations: GSH, reduced glutathione; GSSG, oxidized glutathione; uSt B, unstandardized B value.

## DISCUSSION

The results of the present study showed that the extracellular thiol-disulfide homeostasis balance was shifted toward the oxidative spectrum in both HT and NAIH groups. In contrast, intracellular glutathione homeostasis was comparable between study groups. Additionally, the HT and NAIH groups had significantly higher values of oxidative indicators (GSSG/GSH and GSSG/[GSH+GSH] ratios) and lower values of antioxidant indicator (GSH/[GSH+GSSG]) than the control group. Finally, there was a significantly inverse directional relationship between extracellular thiol levels and anti-TG and TSH levels.

Although serial redox reactions are essential for the synthesis of both thyroid hormones, the connection between thyroid function and redox balance remains unclear (17,18). Thyroid hormones regulate oxidative metabolism and influence free radical production. Changes in thyroid hormone levels affect oxidative stress, with hypothyroidism leading to an increase in oxidants and a decrease in antioxidants (19). A study examining oxidative stress in overt and subclinical hypothyroidism found that oxidative stress, indicated by malondialdehyde, was higher in both conditions, but antioxidant levels were not similarly affected (20). Baskol and cols. reported that malondialdehyde levels were higher after 6 months of thyroxine replacement compared to pretreatment levels, while paraoxonase 1 levels increased after hormone replacement (21). Our findings regarding antioxidant recovery following treatment align with previous studies, which reported that levels of some antioxidants, such as native thiol, increased after levothyroxine replacement but GSH levels remained unchanged.

Autoimmune thyroid dysfunction arises from an immune response against the thyroid. One of the clinical manifestations of autoimmune thyroid diseases is Hashimoto’s thyroiditis, which is characterized by cell-mediated destruction (22). Chronic inflammation can decrease nitric oxide availability in Hashimoto’s thyroiditis, leading to oxidative stress (23). Autoimmune thyroid dysfunction is closely associated with several interleukins and adhesion molecules. Some of these interleukins and adhesion molecules break down the integrity of the thyroid gland and allow the infiltration of T cells and other immune cells into the thyroid tissue (24). Autoimmune conditions are often accompanied by an upregulation of autoantibodies and an imbalance in relevant immune cells. They lead to increased levels of inflammatory cytokines that cause oxidative stress (25). Recent literature reports have shown that interleukins, which are common indicators of systemic inflammation, are increased in Hashimoto’s thyroiditis (23,26). Oxidative stress and inflammation are tightly interlinked, a well-known key factor behind the inflammatory process. As a result, oxidative stress is involved in a variety of pathologies, including inflammatory and immune-mediated disorders (27,28). Current research has thoroughly examined oxidative stress in thyroid hormone abnormalities using a range of oxidative indicators. Advanced glycation end products, for instance, have been studied in a variety of inflammatory and autoimmune illnesses as a predictive sign of oxidative stress. Ruggeri and cols. and Rosaria and cols. have found that advanced glycation end products were significantly higher in patients with Hashimoto’s thyroiditis and that oxidative stress was prevalent even in patients with Hashimoto’s thyroiditis who were euthyroid (29,30). Another study assessing oxidative stress in patients with euthyroid Hashimoto’s thyroiditis *versus* healthy controls found that total antioxidant status (TAS) levels were significantly lower, while total oxidant status (TOS) and malondialdehyde levels were significantly higher in patients with autoimmune hypothyroidism compared with healthy subjects. Additionally, TAS levels correlated negatively with anti-TPO and anti-TG levels (31). Similarly, the results of the present study revealed that patients in the HT and NAIH groups had significant oxidative stress, especially in the extracellular component, compared with healthy controls, and that antioxidant molecules correlated negatively with anti-TPO and anti-TG.

Several studies have evaluated the thiol-disulfide balance in hypothyroidism, with controversial results. A recent study assessing thiol-disulfide homeostasis in subclinical hypothyroidism found that, while native thiol levels and native thiol/total thiol ratios were decreased in patients with subclinical hypothyroidism, disulfide, disulfide/native thiol ratios, and disulfide/total thiol ratios were significantly increased in the subclinical hypothyroidism group compared with a control group. In addition, anti-TPO and anti-TG, correlated positively with the disulfide/native thiol ratio (32). Ates and cols., in a comprehensive study on oxidative metabolism and Hashimoto’s thyroiditis, reported that TOS levels increased gradually from euthyroid to subclinical and overt hypothyroid groups compared with a control group. Total thiol levels increased gradually, from patients with hypothyroidism who were euthyroid to those with overt hypothyroidism, compared with controls. However, the TAS status remained unchanged in patients with euthyroidism and subclinical hypothyroidism and decreased in those with overt hypothyroidism compared with controls (33). In another study, the same authors investigated the effect of levothyroxine therapy in patients with Hashimoto’s thyroiditis and reported that TOS and the oxidative stress index (OSI) decreased in these patients, whereas TAS and total thiol levels increased significantly after treatment. The authors suggested several explanations for the effects of levothyroxine therapy on oxidative stress in Hashimoto’s thyroiditis, such as decreasing proinflammatory cytokine levels and lowering lymphocyte infiltration (34). In a similar manner, we evaluated the effect of levothyroxine therapy in patients with Hashimoto’s thyroiditis and in those with nonautoimmune hypothyroidism. Our results show that treatment with levothyroxine did not cause significant differences in the recovery of oxidative stress in both HT and NAIH groups regarding extracellular thiol-disulfide homeostasis and intracellular glutathione homeostasis compared with the control group.

To the best of our knowledge, the present study is the first one to evaluate oxidative stress in Hashimoto’s thyroiditis by evaluating the intracellular glutathione homeostasis together with the extracellular thiol-disulfide homeostasis. We found that GSH, total GSH, and GSSG levels did not differ significantly between study groups. However, indices of glutathione homeostasis, which indicate dominance of oxidants, were higher and the GSH/(GSH+GSSG) ratio was lower in the HT and NAIH groups compared with the control group, independent of the presence of autoimmunity.

The liver, one of the target tissues of thyroid hormones, is essential for GSH synthesis and GSH-dependent enzymatic antioxidant contents. The effect of decreased levels of thyroid hormones on GSH concentration is controversial. Some studies report higher GSH levels in hypothyroid states, which could result from an adaptive response following thyroid hormone replacement therapy (35-37). However, a recent study revealed that female patients with newly diagnosed Hashimoto’s thyroiditis had significantly reduced serum GSH concentrations than matched control subjects and concluded that decreased GSH levels were associated with immunological response (38). In contrast, our study found no significant differences in GSH concentrations across study groups. Indeed, total GSH and GSSG levels were mildly higher in the hypothyroid groups than in the control group, but this difference was not significant. However, the indices that indicate the direction of the balance, whether to the oxidative or antioxidative spectrum, differed significantly between the HT and NAIH groups in relation to the control group, in which the oxidative markers (GSSG/GSH and GSSG/[GSH+GSSG] ratios) were higher and the antioxidant marker (GSH/[GSH+GSSG] ratio) was lower in the HT and NAIH groups compared with the control group.

The present study has some limitations, including the absence of a large sample. Additionally, the study included only women, since the prevalence of thyroid diseases is higher among them. The absence of participants not receiving hormone replacement is another limitation of the study. Lastly, it is important to note that age affects the levels of native and total thiol. Notably, the mean age in the control group was lower than that in the NAIH group but comparable to that in the HT group.

In conclusion, there was an oxidative dominance in the extracellular space in the HT and NAIH groups compared with the control group. The thiol-disulfide redox balance was shifted toward the oxidative spectrum in the HT and NAIH groups, which had decreased native thiol levels compared with healthy controls. Intracellular glutathione homeostasis – including GSH, total GSH, and GSSG levels – was comparable among the study groups, but the GSH/(GSH+GSSG) ratio was significantly lower and the GSSG/GSH and GSSG/(GSH+GSSG) ratios were significantly higher in both the HT and NAIH groups compared with the control group. These results suggest that hypothyroidism, even after restoration of an euthyroid state, is significantly associated with oxidative stress, independent of the presence of autoimmunity.
